# The Library of the Good Huswife

**Published:** 1894-12-08

**Authors:** 


### The First Century of English Medical Literature.

### X.—THE LIBRARY OF THE GOOD HUSWIFE.

Women were rather awkwardly situated with regard to medicine in Elizabethan days. They were obliged on the one hand to know a good deal; they were vehemently exhorted on the other not to know too much. In their own households and among the surrounding poor, occasions were continually arising which called for the exercise of their skill, but the almost inevitable result was to stir up the opposition and contempt of the regular practitioner, equally disgusted whether they cured or killed. Many of the most learned works on medicine contain bitter complaints of the interference of "presumptuous woman." Members of the faculty felt it to be a grievance that, having bestowed "so much labour and study to reck our brains in reading so many authors," the women with only mother wit to guide them should be allowed to prove serious rivals.

The more discreet housewives treated medicine as a mere branch of cookery and disguised their pretensions to the science under the cloak of devotion to the art. In accordance with this confusion of practice hints on medicine came to be considered a regular part of "Delights for Ladies," "Good Housewives' Treasuries." "Country Gentlewomen's Contentments," and such like female compendiums.

The most attractive of these productions is the "Country Contentments, or English Huswife" (1623), of Gervase Markham, "containing the inward and outward virtues which ought to be in a complete woman." The inward virtues may be summed up as religion and a good temper. Her garments are to be "comely and strong made, as well to preserve the health as adorn the person, altogether without toyish garnishes or the glosse of light colours." She must know the most ordinary fevers and how to keep her household in good health by administering any wholesome receipts or medicines, but on no account must she be too much of a doctor; while her crowning accomplishment must be cookery. "If she is no cook she is only half a wife, and can but perform half her vow; for she may love and obey, but she cannot serve and keep him with that true duty which is ever expected." The housewife is not likely to do much harm with the prescriptions set out for her guidance by Gervase Markham. They are mildness itself. "For apoplexy the strong scent of a fox is exceeding sovereign." For the falling evil a mole—male for a woman, female for a man—is to be dried and drunk in water; and for deafness the housewife is to procure a gray eel with a white belly and bury it alive in an earthen pot in a dung heap for a fortnight. The oil distilled from the eel is then to be dropped into the ear. These fantastics suggestions are, however, the exception. For the most part the housewife is to confine herself to sundry sorts of herb teas, and she is to be sure on any real emergency to call in regular aid.

It is noteworthy that the prescriptions recommended in these manuals for ladies often owe their sole value to some nourishing ingredient in which they were to be administered. And this is specially the case with cure for consumption, a disease as prevalent then as now, to judge from the constant directions for its treatment. For example, the dried ash leys recommended for this malady in the "Ladies' Cabinet" of 1639, can but have proved disappointing, but the ale or broth in which they were to be drunk may well have proved a useful stimulant. Warm mare's milk is recommended in the same manual, a rather exceptional suggestion, as the value of milk was then little understood as an article of diet. Another much-esteemed cure for consumption began with making broth of twenty cocks, and it may reasonably be inferred that no after admixture of foreign bodies could altogether nullify the good effects of so liberal a form of nourishment. Of the same order is a prescription for consumption set forth by Owen Wood in a curious little volume compounded for the benefit of "householders in the country and for the help of such ladies and gentlemen who of charity labour to do good." The "medicine" is composed of six cock sparrows, two wag-tails, hartshorn and ivory, herbs of sorts, spices, bread steeped in malmsey, half a cock, finally six knuckles of veal. The unhappy patient was seldom allowed to take food or drink uncontaminated by drugs. A recipe in "The Good Huswife's Handmaid" (1588), to boil mutton for a sick body, directs the following additions: "A crust of bread, fennell roots, parsley roots, currants, great raisins, and herbs according as the patient is. If he be cold, hot herbs may be born: if he be hot, cold herbs be best, as endive, cinnamon, violet leaves, and some sorrel. Then put in prunes and a very little salt." It does not seem to have occurred to anyone that the "sick body" might have been equally benefited if permitted to take his mutton in a more palatable form and his drugs separate.

The cure of drunkenness was deemed a very desirable secret for ladies, the object being less to create a distaste for strong drink than to confer the power of imbibing it without ill effects. A popular little work, called the "Widow's Treasury" (1595), has several simple remedies to urge for this end, such as to eat the juice of yarrow or the marrow of pork fasting, or to mix fennell or rue with the drink. In "A Closet for Ladies and Gentlemen" (1608) a more elaborate way is given "to help a drunken man or woman for ever." "Give the patient a purgation. And after that give him a swallow to drink in powder for the space of twelve days, and for a month's space give him every day to eat four almonds with four leaves of wood beaten. You must eat them in the morning next your heart."

Swallows were a not uncommon ingredient in old decoctions. In "The Good Huswive's Jewell," by Thomas Dawson, shrunken sinews were to be anointed with oil made as follows: "Take eight swallows ready to fly out of the nest, drive away the breeders when you take them out, and let them not touch the earth; stamp them till the feathers be not perceived, and add lavender cotton, strings of strawberries, tops of mother thyme, rosemary, and May butter."

It will be seen that most of these feminine remedies had at least the merit of being able to do the patient no harm, and if some seem a little inadequate, as, for instance, the advice to staunch bleeding by pricking a great spider and smelling thereto, nature was not impeded in the working out of her own cure.

